# Cancer as a disease of old age: changing mutational and microenvironmental landscapes

**DOI:** 10.1038/s41416-019-0721-1

**Published:** 2020-02-11

**Authors:** Ezio Laconi, Fabio Marongiu, James DeGregori

**Affiliations:** 1 Department of Biomedical Sciences, Section of Pathology, University of Cagliari School of Medicine, 09126 Cagliari, Italy; 2 Department of Biochemistry and Molecular Genetics, Integrated Department of Immunology, Department of Pediatrics, Department of Medicine (Section of Hematology), University of Colorado Anschutz Medical Campus, Aurora, CO 80045 USA

**Keywords:** Cancer microenvironment, Cancer epidemiology

## Abstract

Why do we get cancer mostly when we are old? According to current paradigms, the answer is simple: mutations accumulate in our tissues throughout life, and some of these mutations contribute to cancers. Although mutations are necessary for cancer development, a number of studies shed light on roles for ageing and exposure-dependent changes in tissue landscapes that determine the impact of oncogenic mutations on cellular fitness, placing carcinogenesis into an evolutionary framework. Natural selection has invested in somatic maintenance to maximise reproductive success. Tissue maintenance not only ensures functional robustness but also prevents the occurrence of cancer through periods of likely reproduction by limiting selection for oncogenic events in our cells. Indeed, studies in organisms ranging from flies to humans are revealing conserved mechanisms to eliminate damaged or oncogenically initiated cells from tissues. Reports of the existence of striking numbers of oncogenically initiated clones in normal tissues and of how this clonal architecture changes with age or external exposure to noxious substances provide critical insight into the early stages of cancer development. A major challenge for cancer biology will be the integration of these studies with epidemiology data into an evolutionary theory of carcinogenesis, which could have a large impact on addressing cancer risk and treatment.

## Background

As we consider the question of why we get cancer, it is useful to ask an alternative question: why are we, as humans, so good at not getting cancer for at least 4–5 decades. For example, only 1.7% of all cancer-related deaths in both sexes occur before the age of 40 years in the United States of America, and 90% of cancers are diagnosed in those aged >50 years.^1^ Each adult human comprises roughly 40 trillion cells,^2^ and other animals exist that are far bigger, including the record-holding blue whale with roughly 2000 times more cells. We should therefore marvel at how natural selection has forged mechanisms that allow for the development and maintenance of an enormous number of co-operating cells, for typically over half a century in the case of humans, with life-disrupting malignant growths relegated to later decades. Selection for these mechanisms has been driven by one overriding factor—reproduction. Since cancer almost always led to the death of its host prior to the past century, there has been strong selection to minimise the incidence of cancer through years where individuals were likely to contribute to future generations (through reproduction and postnatal care). For most of our evolutionary history, given extrinsic hazards like predation and pathogens, as well as limited food, most humans did not live to the ages where cancer and other disease of ageing are now prevalent.^3,4^ Medawar, Williams, Hamilton, Kirkwood and others have described how natural selection has invested in the maintenance of our animal bodies (the soma) to the extent that maximises reproductive success,^5–8^ and here we will describe how these same investments limit the incidence of cancer to achieve a similar goal. Hence, both longevity and tumour suppression can be viewed through the lens of life history theory, which encompasses the developmental, reproductive and tissue maintenance strategies that different organisms have evolved to maximise their reproductive success. These evolved strategies are highly dependent on external conditions that influence mortality. For example, investing in long-term tissue maintenance would be energetically wasteful for an organism that undergoes high rates of extrinsic mortality (such as from predation), which would prevent any realisation of benefit (in reproductive output) from such an investment.

There is debate in oncology about whether cancer risk is more dependent on the occurrence of mutations or on changes in tissues that influence somatic evolution, as well as on the relative influence of extrinsic (e.g. smoking) and intrinsic (cell divisions) factors. In terms of extrinsic and intrinsic factors, Tomasetti, Li and Vogelstein have used mathematical and statistical methods to develop a model that explains cancer risk in different tissues based on the number of lifetime cell divisions in stem cells, which, together with external exposures and the influence of inherited genetics, dictates the pace of mutation accumulation and thus cancer risk.^9,10^ Hannun and colleagues generated a model that affixes a much greater role to extrinsic exposures (from cigarette smoking to sun exposure) on the accumulation of mutations and thus cancer risk.^11^ Importantly, both groups attribute the role of all causative factors, whether intrinsic, extrinsic or inherited, to one contributing factor—mutations. Notably, these studies lack consideration for how changes in tissue landscapes with age or exposure to extrinsic factors can influence somatic evolution.^12^ Evolution is driven by environmental changes that result in selection for new phenotypes that are adaptive to the altered landscape. As will be described in more detail below, somatic evolution in our bodies is similarly driven by alterations in tissues, whether by ageing or extrinsic factors, which promote selection for new phenotypes adaptive to altered microenvironments. Some of these new phenotypes can result from oncogenic mutations and epigenetic changes, which have the potential to contribute to the evolution of cancers.

To appreciate how cancer evolves within us, and the mechanisms that have been selected for in animals to suppress this somatic evolution, we need to consider the forces that have driven the evolution of life on our planet. The evolution of the vast diversity of animals, plants and microbes on earth has been driven over several billion years by changing environments, including dramatic shifts in climate, atmospheric gases, extrinsic threats and resource availability. The causes of these changing environments have been varied and include volcanic activity, collisions with extra-terrestrial bolides, tectonic plate movement and life itself.^13^ In some ways, these factors mirror the intrinsic and extrinsic factors contributing to somatic evolution within us. Notably, evolution on earth has not been steady. Periods of relative stasis, where the pace of evolution is much slower due to environmental stability, are punctuated by periods of rapid evolutionary change that follow dramatic changes in the earth’s environment (most notably for the five major extinction events that serve as boundaries between major periods).^14,15^ The pace of evolution changes not because the mutation rate changes but because altered environments change selective pressures. Similarly, changes in tissue microenvironments (landscapes) due to insults or ageing alter selective pressures, dictating the direction of somatic evolutionary change. Changes that occur in tissues include alterations in the fitness of stem and progenitor cells, immune infiltrates, oxygen/nutrient availability, levels of cytokines and growth factors and the extracellular matrix.^16,17^ Such microenvironmental change engenders selection for new adaptive phenotypes, some of which can contribute to cancer development. Mutations (including epigenetic changes) provide phenotypic variability upon which selection can act and are critical contributors to somatic evolution. Increasing rates of mutation should thus enhance somatic evolution. Nevertheless, just as evolution on earth has not been constant, we must consider how the major factors associated with cancer risk, from ageing to smoking to our genetic heritage, influence tissue landscapes and thus the selective pressures acting on cells with mutation-engendered phenotypic change.

In this article, we will describe the mechanisms that we and other animals have evolved to avoid cancer through reproductive years, including by limiting the accumulation of mutations, particularly if these mutations alter cellular phenotypes, and by maintaining youthful tissue landscapes so as to favour the status quo. These mechanisms are achieved through low mutation rates, investments in cellular fitness and effective elimination of cells with damaging or potentially oncogenic mutations/epigenetic changes and involve systems that range from molecular (e.g. DNA repair, proteostasis, autophagy) to tissue level (cell competition) to body wide (the immune system). However, as emphasised above, even if we live idealised lives with plenty of exercise, a balanced diet, and minimal exposure to noxious substances, we will still undergo physiological decline, with an increased risk of cancer and other diseases. Understanding both how we avoid cancer and why we get it will be critical for limiting its impact on our lives.

## Why we age, and why we get cancer in old age

**Fig. 1:**
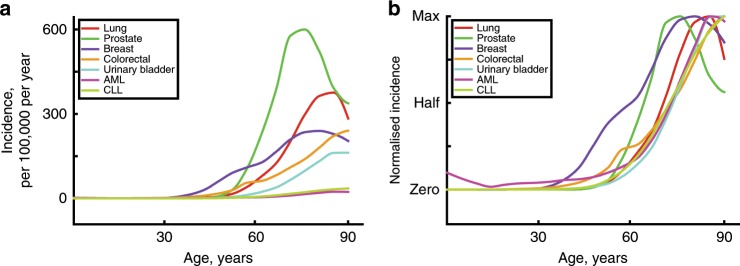
Age-dependent incidence for the most common cancers and leukaemias. The incidence of the five most common cancers (excluding skin cancers) and the two most common leukaemias in the United States from 2012 to 2016. Data are from the National Cancer Institute (www.seer.cancer.gov). **a** Absolute incidence per 100,000 people per year by age. **b** Normalised incidence was derived by first subtracting the minimum value from each value in a data set (to set the minimum to zero on the *Y*-scale) and then dividing the resulting values by the maximal (of those resulting) in the data set (removes vertical scale).

As discussed above, understanding why we get cancer mostly when we are older requires an appreciation for why our tissues decline in old age. The inextricable link between ageing and cancer is highlighted by a simple observation—the incidence curves for most common cancers are strikingly similar, rising after the age of 50 years (Fig. 1), despite the large variance in the numbers of driver mutations evident in these cancers and the fact that they originate in different stem cell pools with large differences in size and organisation.^18^ The current multistage model of carcinogenesis posits that the exponential increase in cancer incidence with age results from the sequential accumulation of oncogenic mutations in a single clone. Indeed, the Cancer Research UK website states that ‘Older age is the main risk factor for cancer. This largely reflects cell DNA damage accumulating over time. Damage can result from biological processes or from exposure to risk factors’.^19^ Mathematical modelling demonstrates that the current multistage model of carcinogenesis cannot easily account for this common incidence pattern across cancers, but these discrepancies can be resolved by the incorporation of ageing-dependent somatic selection and life history-dependent evolution of species-specific tumour-suppressor mechanisms.^18^ Furthermore, the concordance of incidence curves for other diseases of ageing, such as heart and kidney diseases,^20,21^ with the curves for cancer is also consistent with somatic evolution leading to cancer being driven by age-dependent tissue decline. Finally, it is also notable that the shape of incidence curves for lung cancers is very similar for smokers and never smokers, rising sharply after mid-life; although smoking substantially determines who gets lung cancer, age largely determines when these cancers arise^22^ (Fig. 1). Other cancers that are clearly associated with external exposures, such as skin cancers, also show similar late-life exponential increases.^23^ This association with age is quite surprising, as one would anticipate that earlier and greater mutation accumulation and/or tissue landscape changes due to cigarette smoking or sunlight exposure would lead to earlier cancer incidence. Although the solution to this paradox is not clear, we suggest that the tumour-suppressive potential of youth is more potent than previously realised, limiting cancers through half a century of human life even in the face of increased DNA mutations and highly perturbed tissue environments.

As a cautionary note on the above consideration, chemically induced cancers in rodents do develop largely during the first year of life, significantly earlier than the spontaneous emergence of the age-associated, neoplastic-prone tissue landscape in these animals.^24,25^ However, experimental protocols using carcinogens are typically very intense compared to environmental exposures experienced by humans and other species, and they are therefore more likely to override the evolutionary mechanisms designed to reduce the risk of cancer early in life. Finally, there are clear exceptions to the late life pattern of cancers in humans, including early childhood cancers and lymphomas/sarcomas in young adults. Given that such cancers, in the absence of medical intervention, would greatly reduce the odds that the individual could reproduce, we hypothesise three contexts which could account for such exceptions: first, cancers with early life incidence are typically rare (and thus weakly acted on by natural selection); second, some are associated with modern life in industrialised nations (and thus insufficient time and generations have passed to facilitate the evolution of mechanisms to prevent these cancers; e.g. breast cancers^26,27^); and third, some cancers that occur in young people, such as Burkitt’s lymphoma, are caused by pathogens (which possess their own evolutionary agendas to maximise pathogen fitness).^18^

Why has evolution not selected for immortality? There are two straightforward answers to this question: it is either very difficult and/or not favoured strategically. Quality-control mechanisms ensuring the maintenance of functional proficiency at molecular, cellular, tissue and organismal levels are not permanent because the effectors involved are themselves prone to deterioration, fuelling a feedforward process.^7,28^ For example, the exponential loss of adaptive homoeostasis during ageing is inversely correlated with a similar exponential increase in the presence of oxidised proteins in tissues.^7^ As oxidation also influences effectors of proteostasis (e.g. proteasomes), the degradative capacity towards dysfunctional proteins dramatically wanes as we age, and the overall mechanism becomes eventually overwhelmed.^28^ The presence of reinforcing mechanisms to preserve fitness—more efficient purging of dysfunctional molecules and cells leads to better fitness, which in turn reinforces the purging ability—might also explain the non-linearity in the rate of ageing as damaged cells and molecules accumulate, which is reflected in the non-linearity of the increase in risk for cancer and other ageing-associated pathologies. Finally, biological systems are also endowed with resilience—that is, the capacity to react to and recover from acute perturbations in the environment—which can contribute to tissue maintenance through reproductive periods, with the depletion of functional reserves (from intracellular functions to cells to organs) contributing to physiological ageing.

As we have discussed above, natural selection has acted to invest in tissue maintenance only to the extent that maximises organismal fitness (in terms of reproductive success), which varies in different animals dependent on their respective life histories. So even if immortality was theoretically possible, if preventing physiological ageing required investments in tissue maintenance that would have been better spent on early reproductive success, such a strategy would not be favoured (keeping in mind that, for most animals in the wild, including pre-industrial humans, diseases like cancers currently associated with old age are and were not major causes of death).^29^ As one example, experimental models from worms to mice have shown how boosting autophagy, a critical process for recycling damaged organelles and macromolecules, can extend lifespan and reduce ageing-associated pathologies (including cancer).^30–32^ So why have these benefits not driven selection for further enhancements in autophagy? The answer comes down to cost. Mice with enhanced autophagy eat more to maintain the same weight,^32^ which would be a substantial cost in the wild. In all, we can appreciate that there has been minimal selection against cancer and other diseases of old age beyond periods during which contributions to future generations were most likely. Hence, tumour-suppressor mechanisms, whether mediated by the stabilising selection of youthful tissue landscapes or an effective immune system, wane in post-reproductive years. We now turn to some of these mechanisms.

## Mechanisms of tumour suppression

Organismal fitness requires successful reproduction, which itself requires the avoidance of both extrinsic hazards/limitations as well as intrinsic causes of debilitation or death, such as from cancers. Not surprisingly, animals have evolved numerous mechanisms to avoid cancer, which are enforced through a suite of quality-control strategies carried out by cell elimination, hierarchical tissue organisation, immune systems and cell competition. We will also describe how maintenance of youthful tissue landscapes can impede cancer development. These mechanisms work in concert with low error rates for DNA replication and effective DNA repair, which limit (but do not eliminate) the occurrence of cells with oncogenic mutations (and which will not be discussed at length in this review, given extensive coverage in previous reviews; e.g. ref. ^33^).

### Cell elimination

Cells experiencing macromolecular damage or dysregulated growth signals can be eliminated from the replicative cell pool by processes such as apoptosis or cellular senescence, helping to avoid tissue decline and/or the risk of neoplastic disease. The cell type and the nature and intensity of the damage can determine the outcome.^34^ Apoptosis implies cell deletion, and it is a relatively non-disruptive means to deal with dysfunctional cells, provided they can be replaced through the division of neighbouring proficient counterparts.^35^ On the other hand, cellular senescence entails an irreversible cell cycle arrest, with no immediate cell death. As such, it is still very effective as an anticancer barrier, in that it prevents the clonal expansion of altered cells. In fact, mice lacking common effectors of cellular senescence such as p19Arf^36^ or p16Ink4a^37^ are predisposed to cancer. Although mitotically blocked, senescent cells are metabolically active and exert profound effects on the surrounding microenvironment. In the case of short-lived ‘acute’ senescent cells,^34^ beneficial effects such as tumour suppression and tissue regeneration are predominantly favoured,^38^ while longer-lasting ‘chronic’ senescent cells can be detrimental, contributing to tissue dysfunction and fuelling carcinogenesis through different mechanisms.^34^ For example, senescent cells express a senescence-associated secretory phenotype, which includes pro-inflammatory cytokines, and growth and matrix remodelling factors, and chronic exposure to these components can inhibit regeneration and worsen tissue damage.^38^

Senescent cells can be targeted by the immune system, the baseline patrolling activity of which is aimed at implementing quality control in our cells and tissues.^39,40^ For example, the expression of ligands for the natural killer (NK) cell receptor NKG2D, including major histocompatibility complex class I polypeptide-related sequence A and UL16-binding protein 2, is consistently upregulated in oncogene-induced senescence and DNA damage-induced senescence, leading to NK-cell-mediated cell elimination.^41^ Furthermore, senescence surveillance of NRAS^G12V^-induced pre-malignant hepatocytes was found to be dependent upon the activation of the CD4^+^ T cell-mediated adaptive immune response.^42^ Thus both innate and adaptive immune mechanisms can contribute to the clearance of senescent cells. The accumulation of senescent cells during ageing correlates with overall tissue damage,^43^ and notably, the deletion of senescent cells in older mice reduces some pathologies of old age, including cancer, as well as increasing lifespan.^44^

Alterations in chromosome number, known as aneuploidy, can occur during somatic cell divisions, when sister chromatids fail to separate, for example. Studies have shown that aneuploidy almost always reduces cellular fitness, with the extent of reduction in fitness dependent upon the particular chromosome gained or lost (with chromosome losses frequently preventing any cell survival).^45^ Not surprisingly, aneuploid cells are effectively eliminated from most tissues, particularly in highly competitive contexts such as the haematopoietic system.^46^ Notably, it is known that the generation rate of aneuploid cells is high during mammalian foetal development (a period of very rapid cell divisions), but that these aneuploid cells are largely eliminated by apoptosis, preserving the integrity of the individual.^47^ By contrast, aneuploid cells are more likely to persist in extraembryonic tissues, consistent with reduced evolutionary pressure for purging mechanisms (as the fitness of the foetus is preserved). For these reasons, it is important not to take the presence of aneuploid cells simply as indicators of ‘genomic instability’, as these cells could also reflect reduced elimination.

### Hierarchical tissue organisation and immune systems

While an adult human has on the order of 40 trillion cells, the vast majority of these cells have little to no capacity to evolve into a cancer. Most cells in our body are differentiated so as to perform the specific functions mandated for that tissue and have minimal ability to proliferate. Many cells within tissues with high rates of turnover, such as the skin, intestines and the hematopoietic system, are also short-lived. These tissues are maintained by a relatively small number of dedicated stem cells, which divide infrequently to produce short-term multi-potent progenitor cells, which can then further expand to produce lineage committed progenitor cells and eventually fully differentiated cells. This hierarchical organisation of tissues is not only effective for the production of mature cells, both for steady-state maintenance and upon demand (such as following injury or infection); this organisation is also inherently tumour-suppressive.^48^ First, the stem cell pools are small, representing a small target for oncogenic mutation-driven somatic evolution. Second, these stem cells divide infrequently, while the progenitor cells that are committed to differentiation are responsible for much more of the cellular expansion. A mutation arising in such a progenitor will likely end up in short-lived differentiated cells and thus not pose a significant risk. Hierarchical organisation of tissues, maintained by a small number of largely quiescent stem cells, is inherently tumour-suppressive by limiting the numbers of somatic mutations that accumulate in stem cells which would otherwise have the potential to persist for the lifetime of the host.

The immune system also functions as a barrier to cancer development. As described above, NK cells and lymphocytes can mediate the elimination of senescent cells. In addition, the adaptive immune system can not only recognise and eliminate malignant cells with neoantigens, limiting tumorigenesis, but also can select for tumour cells that edit or downregulate these epitopes and thus escape immune elimination.^49^ The immune system is just one component of the varied (but often interconnected) evolved mechanisms to avoid malignancies that would otherwise decrease animal fitness (reproductive success).

### Cell competition

More than 40 years ago, Morata and Ripoll described the process of cell competition in the *Drosophila melanogaster* wing disk.^50^ Whereas flies with only one copy of particular ribosomal protein-encoding genes could develop relatively normally in a homotypic environment, in mosaic contexts such somatic cells would be eliminated during development by surrounding wild-type cells. This cellular purging is mediated by well-conserved signalling and cell recognition pathways, leading to the recognition of ‘loser’ cells by surrounding ‘winners’, with the engulfment of the former by the latter.^51,52^ Interestingly, an array of different mutations, affecting diverse functions, can turn a cell into a ‘loser’.^53^ Subsequent studies showed that reduced activity of MYC and other genes important for cellular growth and division could similarly engender cell competition to eliminate these less competent cells in both flies and mammals. This process appears essential for maintaining tissues during life, and indeed, disruption of cell competition leads to premature ageing, whereas its enhancement promotes greater longevity in flies.^54^ Importantly, additional studies have shown how increased activity of oncogenic pathways can lead to tumours in the absence of wild-type competition but that a normal background will instead bring about the elimination of these transformed cells (in both flies and mammals).^55^ Oncogenic signalling, which can disrupt cell polarity and attachment, leads to forced extrusion from an epithelial monolayer—the normal neighbours literally push the aberrant cell out.^56^ In fact, clones with oncogenic potential or reduced functionality are extruded from normal skin epithelial structures, helping to preserve tissue homoeostasis.^57^ Interestingly, a high-fat diet, which is associated with an increased risk of cancer, inhibited cell-competition-mediated elimination of RasV12-mutated cells in mouse pancreas and intestine.^58^ High-fat-induced inflammation appears to be involved in this effect, as aspirin was able to restore elimination of mutated cells. Aberrant behaviour, whether structural or biochemical, results in clonal elimination or suppression, dependent on the normal neighbouring cells. This last point provides a cautionary note to the vast majority of cancer modelling that has been done in mice using transgenic or gene disruption technology: when most of the cells of a particular type are oncogenically transformed, the physiological ability of normal cells to provide competition or otherwise monitor for aberrancy is lost. Thus the higher susceptibility to cancer development in these model systems could at least in part be an artefactual consequence of reduced clearance of oncogenically altered cells, as would typically occur in animal (including human) tissues on a normal genetic background.

Additional studies in the skin have revealed critical roles for cell competition in maintaining barrier function during mouse development^59^ and for preventing loss of skin integrity with ageing or damage.^60^ During ageing and following exposure to ultraviolet light, damaged basal epidermal stem cells downregulate the expression of the collagen COL17A1, leading to the formation of reduced numbers of hemidesmosomes. As hemidesmosomes are necessary for the firm attachment of the cells to the basement membrane, cells with lower levels of COL17A1 undergo delamination, leading to the presence of fewer differentiated cells such as fibroblasts and melanocytes, thereby promoting skin ageing.^60^ This process of cell competition is again dependent on the presence of undamaged neighbours with normal COL17A1 expression, and the frequency of such cells decreases in old age. It appears that, over time and with increased damage, the efficiency of cellular purging will decrease as more and more neighbouring cells are themselves less fit. Given this feedforward loop (the presence of more damaged cells leading to less efficient purging, leading to even more damaged cells), one would expect a logarithmic pattern of tissue decline, which fits with the acceleration of our physiological impairment in later decades.^61–63^

All of these mechanisms function to effectively reduce the ‘phenotype-altering mutation rate’, by eliminating cells with less functional or potentially oncogenic phenotypes. Thus, although a mutation rate as measured through neutral genetic changes should simply reflect the ability of cells to repair genomic damage and environmental mutagenicity, the accumulation of phenotype-altering mutations (including oncogenic changes) will be highly dependent on the mechanisms that have evolved for cellular elimination and the benefit of such elimination for animal reproduction. For the latter consideration, the age of the animal is highly relevant, as selective pressures for tissue maintenance and tumour suppression wane during post-reproductive ages. This is true at least when the odds of such contributions to future generations was low for most of the evolutionary and natural history of the animal, as would be the case for a 1-year-old mouse or a 60-year-old human. So cell purging or other cell-intrinsic mechanisms (like apoptosis and senescence) will only be selected for to the extent that they improve organismal fitness, and these mechanisms will be differentially selected in different species and for different mutations.^18^

### Youth is tumour-suppressive; old age is clonogenic

**Fig. 2:**
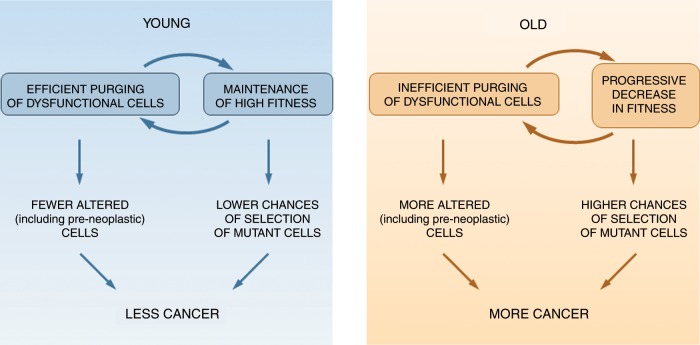
How differences in age-related tissue landscapes influence cancer. Quality-control mechanisms ensure maintenance of cell fitness throughout reproductive ages (YOUNG; left panel). This maintenance results both from efficient purging of altered cells and from a low probability of their selection in a young and healthy tissue landscape. These two mechanisms reinforce each other. By contrast, quality-control mechanisms wane in post-reproductive ages (OLD, right panel). This decline implies that more altered cells accumulate and there is an increased likelihood for their selection when clones possess mutations that are adaptive in the aged tissue landscape. Again, inefficient elimination of altered cells and reductions in cellular fitness reinforce each other.

When we think of evolution, we of course focus on change. But a critical feature of natural selection is the maintenance of useful features. The elimination of mutations that reduce organismal fitness, known as purifying selection, is key to the maintenance of fitness. As such, the conservation of gene sequences across time is a strong indicator of their importance. Although we have learnt a great deal about how organisms have adapted to changing environments, we must equally consider how species can often remain relatively unchanged for many generations, sometimes across millions of years.^14^ In a constant environment, selection will act to favour trait values that maximise fitness, and once those traits are optimised, stabilising selection will act to maintain these traits within this optimised range. Thus, as long as environments remain relatively static, stabilising selection will suppress evolutionary change. We have proposed that similar stabilising selection dominates our somatic tissues, at least when we (and other animals) are young and healthy.^48,64^ Basically, we posit that humans and other animals have evolved stem/progenitor cells together with tissue niches that promote near optimal fitness of these cells. The relatively static nature of tissues through years of likely reproduction favours the ‘evolved type’ of stem and progenitor cells, such that mutations (including oncogenic mutations) that change phenotype are likely to reduce cellular fitness, leading to the elimination of these mutations from the self-renewing cell pool. Indeed, when oncogenic mutations were engineered into haematopoietic stem cells of mice, and their impact assessed under relatively unperturbed contexts, these changes almost always resulted in reductions in stem cell self-renewal, which should result in clonal elimination.^65^ From this perspective, we can appreciate that the mechanisms described above to maintain tissue fitness will also be potently tumour-suppressive, by suppressing phenotypic change in stem cell populations (Fig. 2, left).

However, although we may wish otherwise, we do not stay young forever, as natural selection only acted to invest in tissue maintenance (and ‘youthfulness’) for long enough to maximise reproductive success (given the trade-offs for such an investment). As our tissues undergo physiological decline in old age, selective pressures dictating the strength and direction of somatic evolution will change.^48,66^ Although not the focus of this review, exposure to noxious substances (such as cigarette smoking), diet, exercise or inherited genetics should similarly influence tissue landscapes and thus the trajectories of somatic evolution. Alterations in tissues away from the evolved type will result in selection for genetic and epigenetic changes that are adaptive to this new tissue environment (Fig. 2, right). Indeed, the same oncogenic mutations in mouse haematopoietic progenitor cells that are not selected for in young haematopoietic environments can be strongly selected for in aged bone marrow environments.^67–69^ Moreover, lung tumorigenesis initiated by activating KRAS mutations is enhanced in aged mice relative to young.^70^ Similarly, the livers of old rats promote the expansion of both normal and premalignant hepatocytes to a much greater extent than in young rats,^25,71,72^ and this effect can be counteracted by caloric restriction or time-restricted feeding.^73,74^ The stabilising selection of youth therefore seems to be replaced by positive selection for mutation-induced phenotypic change in old age. The critical role of the microenvironment in dictating such somatic evolutionary trajectories is emphasised by the demonstration that blocking inflammation, a known contributor to ageing-associated tissue decline and some cancers, can prevent the ageing-associated selection for these oncogenic mutations.^67^ So, although we might not be able to avoid many, if not most, of the mutations that occur throughout life, we can, in theory, alter the strength and direction of selection acting upon the mutated cells by modulating tissue environments. For the different examples given above, decreased somatic cell fitness (in terms of cell cycling, survival or maintenance of self-renewal) of normal competing cells appears to be key in allowing the competitive expansion of pre-malignant cells.^75,76^ In fact, increasing the fitness of normal competitors can suppress the expansion of malignant clones.^77–80^ For example, the infusion of normal young hepatocytes—a source of cells with a high competitive fitness—into the livers of rats exposed to a protocol for the induction of hepatocellular carcinoma delayed the growth of nodules and their progression to cancer.^71^ In all, we can appreciate how older tissues can be clonogenic to both young, normal and pre-neoplastic homotypic cells.

## Somatic evolution—beyond cancer

The presence of clones of altered cells has been typically associated with (pre)-neoplastic disease states. Focal proliferative lesions in solid tissues (e.g. polyps, nodules, papillomas) or aberrant clones of haematopoiesis are known risk factors for cancer.^81,82^ However, an increasing number of studies have now reported that clones of cells, often with mutations classified as oncogenic, are very common in phenotypically normal human tissues as we age, to the point that they were proposed to be considered as a hallmark of ageing.^81,83–90^ In particular, analyses of sequencing data from peripheral blood, oesophageal epithelium, and sun-exposed skin have revealed abundant clones with known oncogenic-driver mutations (reviewed in ref. ^91^). These clones increase in frequency in old age, often in a logarithmic pattern that parallels cancer risk. By necessity, such findings call for a critical reappraisal of the biological significance of aberrant clonal expansions.^92^ Why do they emerge in aged tissues? Importantly, data suggest that the driving force for clonal growth is selection, as opposed to random drift^90,93^ (but see ref. ^94^), implying a fitness advantage in a defined tissue landscape. Indeed, the presence of oncogenic mutations (such as mutations that lead to premature termination in tumour-suppressor genes) substantially exceeds expectations based on chance (e.g. refs. ^90,93,95^). Some of the experimental results described above suggest that the pervasive presence of clonal expansions as we age is a biological result of the emergence of more competitive cells in a background of declining functional proficiency. Basically, oncogenic mutations can be adaptive for cells within aged tissue microenvironments, even though these same mutations provide much less (or no) selective advantage in healthy young tissues.

Of course, our lifestyles and exposures greatly influence our cancer risk. Major risk factors of human neoplasia, including smoking, alcohol and ultraviolet light, are increasingly being considered for their impact on the tissue microenvironment. For example, clones of p53-mutant keratinocytes were found to be both more frequent and larger in sun-exposed human skin compared to sun-shielded areas,^96^ suggesting that sunlight could increase selection for p53-deficient cells. As shown for the haematopoietic system and oesophageal epithelium, clones harbouring a particular constellation of oncogenic mutations have been shown to be associated with exposures linked to increased cancer risk, including smoking, alcohol consumption and chemotherapy/radiation therapy.^85,97–102^ The nature of the selective environment is crucial to the genotype/phenotype of the clone that emerges, as one would expect.^85^ In the haematopoietic system, age-related clonal expansions involving TP53 and spliceosome genes are predictive of the later development of acute myeloid leukaemia,^103^ and clones with TP53 and PPM1D mutations are observed in patients treated previously with chemotherapy and radiotherapy.^98,99,100^ We can surmise that tissue landscapes that favour particular mutations (such as in TP53) are conducive to oncogenesis and that particular insults mediate selection for high-risk mutations.

**Fig. 3:**
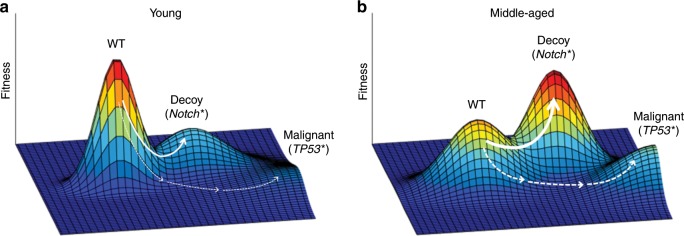
Decoy fitness peaks and tumour suppression. **a** Hypothetical evolutionary fitness landscapes depict the relationships between genotype/epigenotype and fitness (shown here for somatic cell fitness). The *x*–*y* plane represents potential genetically and epigenetically encoded somatic cell phenotypes. Genetic changes can either decrease (downhill) or increase (uphill) fitness. We have previously proposed that on a young fitness landscape ‘wild-type’ (WT) cells occupy a high peak, as evolution over millions of years has optimised stem and progenitor cell adaptation to their tissue niche.^48^ Thus phenotypic change resulting from genetic or epigenetic mutations will mostly result in cells with reduced fitness, thus disfavouring evolution towards malignancy. We further propose that some somatic mutations (such as in Notch1) can create ‘decoy peaks’, which confer low risk of further progression to cancer. Progression up the decoy peak may be limited by the required passage through lower fitness intermediates, but the small size of epithelial progenitor pools could facilitate such transitions through neutral drift. Alternatively, a single mutation, such as in Notch1, could mediate the ‘jump’ to the other peak. **b** By middle age, fitness landscapes engender greater selection for the phenotypes that occupy decoy peaks (often with Notch1 mutations); while partially transformed, cells on these decoy peaks are more benign with reduced malignant potential. At older ages, further tissue degradation and damage accumulation should result in a landscape that increases the odds of mutational adaptation towards both benign (decoy) and more malignant phenotypes. Arrow thickness reflects hypothetical probabilistic phenotypic and fitness effects of mutation. Note that for simplicity this model does not incorporate roles for ageing-dependent mutation accumulation, which should clearly contribute to cancer risk with age. We also note that, while experimental and observational data support changes in somatic selection in aging tissues, the shapes of these landscapes are hypothetical. Figure and legend were modified from Higa and DeGregori^104^ under a CC-BY licence.

In the oesophagus, clones with TP53 mutations are associated with smoking and alcohol consumption^85^; notably, these clones largely arise in the elderly, suggesting that youth can exert a suppressive effect on these expansions (despite smoke and alcohol exposures). In fact, while low-dose radiation led to selection for TP53 disruption in the oesophageal epithelium of mice, dependent on oxidative stress, TP53 loss was not selected for in healthy young epithelium.^78^ Selection for TP53 loss is clearly context dependent and, importantly, can be pharmacologically controlled. Interestingly, clones with certain mutations (most notably, damaging mutations in the NOTCH1 gene) arise earlier in life than TP53 mutations: clones driven by NOTCH1 mutations arise by the age of 40–50 years, whereas clones with inactivating TP53 mutations arise substantially later.^85,93^ Reduced Notch1 function appears to be adaptive in oesophageal epithelial cells by middle age, in that Notch1 loss appears to provide a selective advantage to oesophageal cells at earlier ages than those driven by TP53 mutations (or even oncogenic mutation-driven clonal haematopoiesis). Notably, while inactivating Notch1 mutations constitute more than half of the epithelial content of individuals over 50 years, such mutations are much less prevalent (~10%) in oesophageal carcinomas.^85,93^ One speculative idea is that adaptation via Notch1 inactivation allows epithelial progenitors to occupy fitness peaks (dubbed ‘decoy fitness peaks’) that might reduce the selective advantage conferred by more malignant mutations such as in TP53 (and thus progression of progenitors up malignant fitness peaks) (Fig. 3).^104^ Basically, Notch1 mutations improve the fitness of progenitor cells within the age-altered oesophageal epithelium, resulting in a cell pool that is less likely to be further improved by additional cancer-promoting mutations (high somatic fitness, *when non-malignant*, can be tumour-suppressive). We can thus appreciate that somatic evolution per se is not selected against by natural selection, but somatic evolution that can reduce organismal fitness is. Indeed, in the liver, selection for clones with mutations that are adaptive in a cirrhotic liver has been shown to contribute to improved liver function and regeneration.^105^

## Potential therapeutic opportunities

**Fig. 4:**
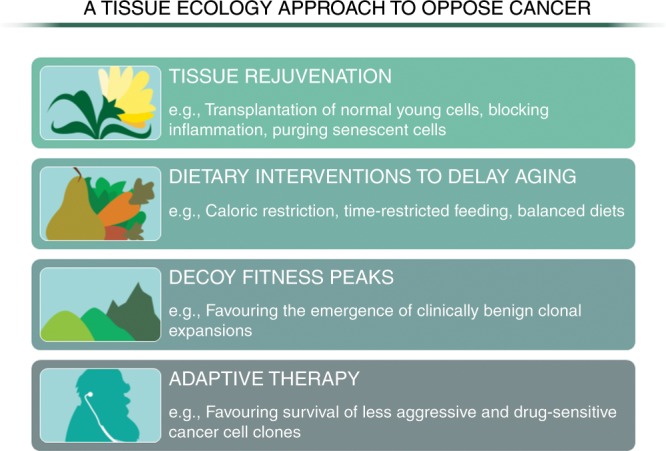
Strategies to target neoplastic-prone tissue landscapes. Various evolution-informed strategies can be envisioned to limit cancer incidence and to improve treatment outcomes. For cancer prevention, the ideas and evidence presented here indicate that the selection for malignant clones can be countered by the preservation of a younger tissue landscape (e.g. through dietary interventions, reducing age-associated inflammation). In addition, while more speculative, interventions that mimic decoy fitness peaks, such as by providing adaptive but non-malignant changes to cells in aged tissues, could potentially delay cancer evolution. For therapeutic interventions for the treatment of a metastatic cancer, recent studies indicate the ability of less aggressive and/or more therapy-sensitive cancer cell populations to limit the dominance of more malignant and therapy-resistant clones, which are associated with a worse prognosis.

From our discussion so far, we can envision evolutionary theory-informed strategies to limit or control cancers (Fig. 4), such as through the rejuvenation of tissue landscapes (e.g. transplantation of young cells) or blocking ageing-associated changes in the microenvironment (e.g. eliminating senescent cells or reducing inflammation). For example, rapamycin was reported to increase health span or even reverse ageing-related alterations in several species, from worms to dogs.^106,107^ Similarly, several senolytic drugs (i.e. agents able to clear senescent cells) are being proposed to counter the rate of ageing and cancer development).^108,109^ However, such approaches need not involve drugs, given the demonstrated abilities of good (and limited) diet and exercise to extend lifespan and limit tumorigenesis.^78,79^ Among multiple potential mechanisms, dietary restriction delays tissue aging via improvement of proteostasis and protection of the endoplasmic reticulum hormetic response, thereby extending the efficiency of protein quality-control mechanisms and decreasing protein misfolding.^110^ Like caloric restriction, exercise promotes increased autophagy and improved proteostasis,^111,112^ augmenting tissue maintenance, and reduced chronic inflammation,^113^ all of which (as described above) should decrease tumorigenesis. As another implication of ideas presented in this review, interventions to favour decoy fitness peaks (such as NOTCH1 mutant clones in the oesophagus) could limit or delay the emergence of more malignant clones.^104^ Moreover, evolutionarily informed strategies for cancer treatment, such as adaptive therapy, which leverage competition from drug-sensitive cells to keep a cancer treatable, are also being developed.^114,115^ Traditionally, patients are treated with the goal of eliminating the most cancer burden possible, with treatment to the maximum tolerated dose. Most patients with advanced cancer will relapse with drug-resistant disease. With adaptive therapy, patients are treated only to the extent that tumour burden is reduced but not eliminated, thus better maintaining drug-sensitive cells that provide competition with drug-resistant cells during treatment holidays.

## Concluding remarks

Both cancer and ageing remain two biological processes whose complexity has thus far defied either simple explanations or simple solutions. However, several findings in these fields seem to converge towards basic common aspects and begin to shed light on the mechanisms behind the long-standing association between ageing and neoplastic disease. The pervasive presence of aberrant clonal expansions in aged tissues establishes a direct biological link between the aged phenotype and cancer risk, given the pre-neoplastic nature of some of these clones. In the main article, we have argued that the clonogenic propensity of aged tissues relates to three factors. First, tissue microenvironments decline in old age, given insufficient investment in their maintenance during traditionally post-reproductive periods; second, there is an initial progressive decline in competitive fitness of the bulk of the tissue; this fuels, third, a concomitant relative inability to clear altered (dysfunctional) cells. These alterations synergise in a feedforward mechanism, which is highly likely to select for the emergence of altered cell clones with adaptive mutations, some of which can be oncogenic. This understanding of the forces contributing to cancer genesis and development highlights the potential for evolution-minded approaches for both cancer prevention and therapy.

An important goal for future studies will be the development of interventions, such as those modulating inflammation, autophagy and/or senescent cell accumulation, that impede or at least delay cancer evolution through the maintenance of tissue landscapes that favour normal cells. In addition, the biological significance of ageing-associated clonal proliferations needs to be addressed within an evolutionary framework, taking into account the phenotypic features of the surrounding tissue landscape and with further extension to the whole organism. A most pressing issue pertains to the relationship of these clones with (pre)-neoplastic lesions. In a medical environment that is more and more geared for early diagnosis with ever more sophisticated molecular tools, it is imperative that we better gauge the risks associated with mutant clones and how to mitigate this risk.

## Data Availability

Not applicable.
